# Recurrence Patterns After Complete Cytoreduction for Advanced Ovarian Cancer: Robotic Versus Open Surgery

**DOI:** 10.3390/curroncol33020071

**Published:** 2026-01-26

**Authors:** Yossi Tzur, Yoav Brezinov, Tomer Bar-Noy, Amber Yasmeen, Melica Nourmoussavi Brodeur, Shannon Salvador, Walter H. Gotlieb, Susie Lau

**Affiliations:** 1 Department of Gynecologic Oncology, Jewish General Hospital, McGill University, Montreal, QC H3T1E2, Canadayoav.brezinov@mail.mcgill.ca (Y.B.);; 2 Lady Davis Institute for Cancer Research, Jewish General Hospital, McGill University, Montreal, QC H3T1E2, Canada

**Keywords:** cytoreduction, surgical procedures, laparotomy, neoplasm recurrence, ovarian cancer, robotic surgical procedures

## Abstract

Background: Complete cytoreduction remains the primary surgical objective in advanced ovarian cancer and concerns persist that robotic surgery may result in distinct recurrence patterns and worse oncologic outcomes due to technical limitations, such as the use of pneumoperitoneum, the restricted visualization, and the lack of tactile evaluation. Methods: We retrospectively identified 125 consecutive patients with advanced epithelial ovarian cancer who attained the best outcome surgery can achieve, and compared the outcome of these patients based on whether complete cytoreduction was achieved by laparotomy or robotic surgery. The primary objective was to compare recurrence patterns and secondary analyses assessed perioperative, postoperative, and survival outcomes. Results: Among 125 patients who had complete cytoreduction (78 robotic; 47 open), baseline characteristics were largely comparable, except for higher rates of interval cytoreduction (84.6% vs. 61.7%, *p* < 0.01) and more PARP inhibitor use (19.2% vs. 6.4%, *p* = 0.048) in the robotic group. Recurrence patterns did not differ. Operative and postoperative outcomes were likewise comparable. Median disease-free and overall survival were longer in the robotic group. Conclusions: Among patients achieving complete cytoreduction, recurrence patterns and oncologic outcomes were comparable between robotic and open surgery, suggesting that the route of attaining complete cytoreduction did not influence the outcome.

## Simple Summary

Robotic-assisted cytoreductive surgery is increasingly used in advanced ovarian cancer, but concerns remain about whether this minimally invasive approach affects the recurrence patterns compared to open surgery. This study compared the recurrence patterns and oncologic outcomes of patients with advanced epithelial ovarian cancer who achieved the best outcome attainable by surgery, namely, no residual disease. Oncologic outcomes were comparable between the two approaches, and, among those who recurred, the anatomical distribution of recurrence did not differ between robotic and open surgery. These findings suggest that robotic-assisted cytoreduction can be a safe and oncologically sound option in carefully selected patients who attain no residual disease.

## 1. Introduction

Complete cytoreduction is a cornerstone in the management of advanced ovarian cancer and is consistently associated with improved survival outcomes [1,2]. Over the past decade, neoadjuvant chemotherapy (NACT) followed by interval debulking surgery (IDS) has become increasingly utilized as an alternative to primary debulking surgery (PDS), supported by randomized trials showing reduced perioperative morbidity with comparable survival outcomes [3,4,5]. Consequently, the proportion of patients undergoing NACT-IDS consistently increases and now approaches half of all advanced-stage cases in some centers [6].

Concurrently, minimally invasive approaches for cytoreduction, particularly robotic-assisted cytoreductive surgery, have seen significant adoption [7,8]. These approaches offer surgical de-escalation with potential advantages, such as reduced blood loss, shorter hospitalization, and faster recovery [9]. However, concerns persist regarding the oncologic adequacy of robotic surgery, particularly with respect to upper abdominal visualization, lack of tactile feedback, and pneumoperitoneum effects, which may theoretically limit the completeness of cytoreduction. Importantly, even among patients who achieve complete cytoreduction (R0), the majority with advanced epithelial ovarian cancer have aggressive tumor biology and ultimately recur [10,11,12]. Once all visible disease has been surgically excised, tumor biology might be more important than the surgical approach that was used.

In this context, we aimed to evaluate whether the robotic approach led to distinct anatomic recurrence patterns and oncologic outcomes compared to open surgery in patients who achieved R0.

## 2. Materials and Methods

### 2.1. Study Design, Setting, and Patient Selection

**Figure 1:**
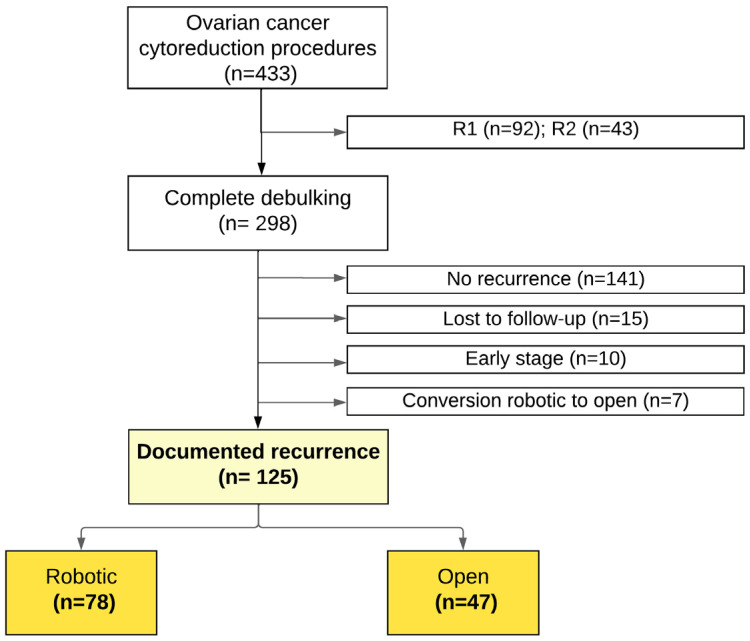
Study cohort flow diagram. *R*1 = macroscopic residual disease < 1 cm; *R*2 = macroscopic residual disease > 1 cm.

This retrospective cohort study was conducted at a tertiary, academic gynecologic oncology center in Montreal, Québec, Canada, following approval from the Institutional Review Board (protocol number MBM-CR18-07). Patient consent was waived due to the retrospective design of the study. Patients diagnosed with advanced epithelial ovarian cancer who underwent cytoreductive surgery between January 2006 and December 2022 were identified through electronic medical records. Inclusion criteria encompassed patients who achieved complete cytoreduction and had documented disease recurrence (Figure 1). Recurrence was defined as the appearance of new lesions or enlargement of previously resolved lesions on imaging, or clinical evidence of disease, in accordance with RECIST 1.1 criteria [13]. Patients without documented recurrence were considered recurrence-free through their last recorded follow-up and were censored at the date of last clinical assessment and/or imaging confirming no evidence of disease.

### 2.2. Surgical Approach

**Figure 2:**
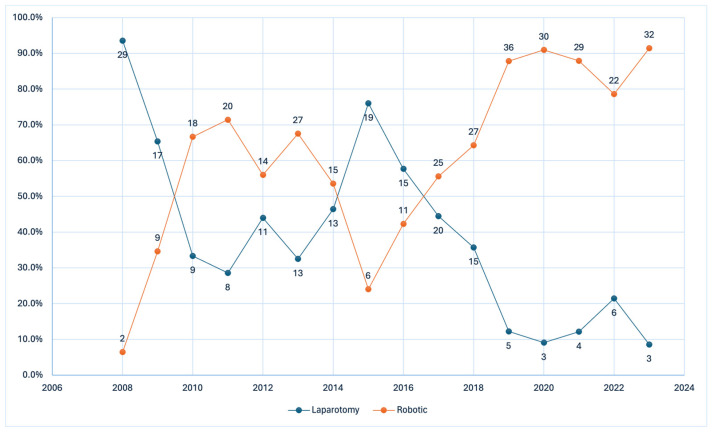
Temporal trends in surgical approach. Proportion of robotic-assisted (orange line) and open (blue line) cytoreductive surgeries performed annually between 2006 and 2022, reflecting progressive adoption of robotic surgery and decline in laparotomy use.

Cytoreductive procedures were performed via either robotic-assisted cytoreductive surgery or open cytoreductive surgery. Treatment approach (robotic vs. open surgery) was determined by a multidisciplinary tumor board based on preoperative assessments, including physical examination, computed tomography imaging, patient performance status, and surgical era (Figure 2). Robotic surgery was considered when disease distribution and operative complexity were deemed potentially amenable to a robotic approach, acknowledging that the ability to achieve complete cytoreduction could not be known preoperatively. To focus specifically on recurrence patterns among patients achieving no gross residual disease, the analysis was restricted to patients who ultimately achieved complete cytoreduction.

### 2.3. Treatment and Follow-Up

All patients were administered standard chemotherapy comprising carboplatin and paclitaxel, according to institutional and guideline standards. Patients undergoing primary debulking surgery (PDS) received adjuvant chemotherapy, typically for six cycles. Patients treated with neoadjuvant chemotherapy (NACT) received 4 cycles prior to interval debulking surgery. This was consistent across the cohort and was determined according to institutional protocols. After surgery, 3 adjuvant postoperative cycles were provided. NACT followed by IDS was offered when complete cytoreduction was considered unlikely with primary cytoreductive surgery.

Homologous recombination deficiency (HRD) status was determined using the myChoice^®^ CDx assay (Myriad Genetics, Salt Lake City, UT, USA), which incorporates a genomic instability score (GIS) based on loss of heterozygosity, telomeric allelic imbalance, and large-scale state transitions. A GIS ≥ 42 was used to define HRD positivity. BRCA mutation status was defined as the presence of a pathogenic BRCA1 or BRCA2 alteration detected through germline and/or tumor testing. Maintenance poly(ADP-ribose) polymerase (PARP) inhibitor therapy, when indicated, has been increasingly adopted in recent years.

Although Hyperthermic Intraperitoneal Chemotherapy (HIPEC) has emerged as a treatment option following interval debulking surgery in selected patients with stage III disease, it was not routinely adopted at our institution during the study period, and therefore was not administered to patients included in this cohort. Additionally, Bevacizumab was used infrequently in this cohort, reflecting lack of provincial reimbursement during the study period.

Patients were followed every 3–4 months for the first two years post-surgery, every six months for the subsequent three years, and annually thereafter. Follow-up evaluations included clinical history, physical examination, and serum CA-125 measurements. Imaging studies were performed as indicated according to new or worsening clinical symptoms (e.g., abdominal pain, bloating, ascites, and weight loss) or new suspicious findings on physical exam. Patients without recurrence or death were censored at the date of last clinical contact. Loss to follow-up was defined as the absence of clinical, laboratory, or imaging follow-up for more than 24 months, and such patients were excluded from the cohort.

### 2.4. Outcomes

The primary outcome was the anatomical pattern of first recurrence following R0 cytoreduction. Recurrence sites were categorized as pelvic, supra-pelvic, retroperitoneal, parenchymal, or extra-abdominal, following the classification scheme of the DESKTOP III trial [14]. Secondary outcomes included intraoperative and postoperative complications, disease-free survival (DFS), and overall survival (OS). DFS was defined as the time from initiation of primary treatment to first documented recurrence or death from any cause. Median follow-up time was calculated using the reverse Kaplan–Meier method based on full available follow-up information. Intraoperative complications were defined as bladder or bowel injury and major bleeding requiring transfusion of ≥2 units of red blood cells. Postoperative complications included wound dehiscence, myocardial infarction, deep vein thrombosis, and wound infection. Chemotherapy Response Score (CRS) was not evaluated for most patients in this cohort, as it was not routinely incorporated into pathology reporting during the majority of the study period.

### 2.5. Statistical Analysis

Categorical variables were compared using Chi-square or Fisher’s exact tests. Continuous variables were compared using two-sided *t*-tests for normally distributed data or Mann–Whitney U tests for non-normally distributed data. Multivariable logistic regression was employed to adjust for potential confounders. Overall survival (OS) and disease-free survival (DFS) were analyzed using Kaplan–Meier survival curves with log-rank tests and Cox proportional hazards models. OS and DFS were calculated from the date of initiation of primary treatment (PDS or NACT) to the date of recurrence, death, or last follow-up. Statistical analyses were performed using IBM SPSS Statistics, version 29.

## 3. Results

### 3.1. Patient Characteristics

During the study period, 433 patients underwent cytoreductive surgery. Of these, 125 experienced recurrence after achieving complete cytoreduction, including 78 (62.4%) who had robotic-assisted surgery and 47 (37.6%) who had open surgery. The median follow-up for the entire cohort was 91 months (IQR, 53–112 months). Patients without documented recurrence during the available follow-up were not included in the analysis (Figure 2). Demographic and baseline clinical characteristics were comparable between groups (Table 1). Patients in the robotic group more frequently underwent neoadjuvant chemotherapy and PARP inhibitor maintenance. Median CA-125 levels at diagnosis were similar between the robotic and open cytoreduction groups (670 U/mL [IQR 333–2128] vs. 643 U/mL [IQR 243–1950], *p* = 0.53).

### 3.2. Recurrence Patterns

The most frequent site of recurrence was the supra-pelvic region in both groups (72.3% vs. 57.7%, *p* = 0.10). Recurrence rates were similar between groups for intrapelvic (44.7% vs. 42.3%, *p* = 0.80) and retroperitoneal sites (34.0% vs. 33.3%, *p* = 0.94). Rates of parenchymal, abdominal wall, and extra-abdominal recurrences were also comparable between groups (Table 2). Among the robotic cases, one recurrence occurred at a prior port site, together with other intraperitoneal disease. Overall, at the time of recurrence, 93 of 125 patients (74.4%) had involvement in multiple anatomic sites, while 32 patients (25.6%) experienced recurrence at a single anatomic site. Among the latter, 24 (75.0%) recurred within the abdomen or pelvis, whereas 8 (25.0%) had isolated extra-abdominal metastasis. A multivariable logistic regression adjusting for age, ASA score, NACT, and PARP inhibitor treatment confirmed comparable recurrence patterns between the two groups (Table 3).

### 3.3. Adverse Events and Survival Outcomes

**Figure 3:**
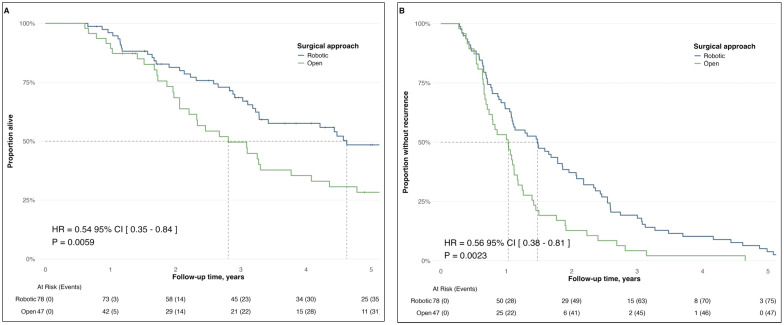
Kaplan–Meier estimates of survival outcomes. (**A**) Overall survival by surgical approach, measured from initiation of primary treatment (primary cytoreduction or neoadjuvant chemotherapy) to death or last follow-up. Median OS was 55.5 months in the robotic group versus 33.7 months in the open group (log-rank = 5.45, *p* = 0.006). (**B**) Disease-free survival was measured from treatment initiation to recurrence, showing median DFS 17.7 vs. 12.4 months for robotic and open groups, respectively (log-rank = 9.5, *p* = 0.002). Vertical ticks indicate censored data.

Intraoperative complications were rare and similar between groups. Postoperative complications were reported in four patients (8.5%) in the open group and in one patient (1.3%) in the robotic group (*p* = 0.07). Lung lesions were the most common site of extra-abdominal relapse (4.3% open vs. 6.4% robotic; *p* = 0.65). Median overall survival (OS) was significantly longer in the robotic group compared with the open group (55.5 vs. 33.7 months; HR = 0.54, 95% CI 0.35–0.84; *p* = 0.006) (Figure 3A). Disease-free survival (DFS) was also longer in the robotic group (17.7 vs. 12.4 months; HR = 0.56, 95% CI 0.38–0.81; *p* = 0.002) (Figure 3B).

## 4. Discussion

In this retrospective cohort of patients with advanced epithelial ovarian cancer who achieved complete cytoreduction (R0), recurrence patterns were comparable following robotic-assisted and open cytoreductive surgery. These findings suggest that once R0 is achieved, the surgical access route does not substantially influence where relapse develops. In carefully selected patients in whom complete cytoreduction is feasible, theoretical concerns related to robotic surgery—such as pneumoperitoneum, limited tactile feedback, and restricted visual access—do not appear to translate into meaningful differences in recurrence patterns. In contrast, one would have expected that these limitations to correctly evaluate R0 in the robotic group would have been accompanied by more recurrences and worse outcomes.

Prior studies evaluating recurrence patterns after minimally invasive cytoreduction have been limited, often with small sample sizes and short follow-up. The MISSION feasibility study included 30 patients with a median follow-up of 10.5 months [15], likely underestimating recurrence distribution. In contrast, recurrence patterns in the open surgery arm of the DESKTOP III trial demonstrated abdominal-pelvic relapse predominance with retroperitoneal and parenchymal involvement [14], closely mirroring the pattern observed in our cohort. This concordance supports the interpretation that recurrence patterns following complete cytoreduction are largely dictated by tumor biology rather than surgical access route.

Importantly, the present cohort represents a highly selected population in whom complete gross resection was frequently achieved with relatively limited surgical effort. Fewer than 10% of patients undergoing robotic cytoreduction required procedures beyond hysterectomy, bilateral salpingo-oophorectomy, and omentectomy. This surgical profile differs from several landmark cytoreductive trials, such as SCORPION, in which high-complexity procedures—including bowel and upper abdominal resections—were common [4]. Accordingly, our findings are most applicable to patients with low-volume disease, in whom complete cytoreduction can be achieved with minimal surgical complexity, and should not be extrapolated to higher-complexity cytoreductive settings.

The major strength of this study is the exclusive focus on patients achieving complete cytoreduction, which represents the best attainable outcome with surgery. Notably, theoretical limitations of robotic surgery, including decreased visualization of some areas of the abdomen and the lack of tactile evaluation, would be expected to overestimate the achievement of complete cytoreduction in the robotic group and, if anything, bias outcomes toward worse results.

This study is limited by its retrospective design and inherent selection bias. Although the analysis was restricted to patients who achieved complete gross resection, this outcome is only known postoperatively and therefore does not eliminate selection bias related to the preoperative choice of surgical approach. Formal measures of surgical complexity, such as the Aletti score [16], and objective assessments of preoperative disease burden were not systematically captured. Although rates of bowel and upper abdominal resections were low in both groups, unmeasured differences in disease burden or operative complexity not reflected by these surrogate markers may have contributed to the observed survival differences. While disease-free and overall survival were longer in the robotic cohort, survival outcomes were not evaluated using multivariable survival models and therefore cannot be attributed to surgical approach. The prognostic impact of neoadjuvant chemotherapy itself remains controversial, with randomized trials demonstrating comparable survival when complete cytoreduction is achieved [4,17]; therefore, survival differences in this study should not be interpreted as reflecting the neoadjuvant strategy. These differences likely reflect temporal and treatment-related factors, including greater use of PARP inhibitor maintenance in more recent years. Finally, HIPEC was not utilized in this cohort, reflecting institutional practice patterns, and the findings may not be generalizable to settings where HIPEC is routinely incorporated.

In summary, among selected patients with advanced ovarian cancer who achieve complete cytoreduction with limited surgical complexity, recurrence patterns were comparable between robotic and open surgery. These findings support the concept that anatomic patterns of relapse are primarily biology-driven rather than access-driven. Accordingly, robotic cytoreduction may be considered an acceptable approach in appropriately selected patients when complete gross resection is feasible. Prospective trials, including ongoing minimally invasive cytoreduction studies, such as LANCE [18] and MIRRORS [19], will be essential to further define the role of minimally invasive surgery in advanced ovarian cancer.

## Figures and Tables

**Table 1:** Baseline characteristics.

	Open (n = 47)	Robotic (n = 78)	*p*-Value
Age (years)	64.6 ± 11.0	64.0 ± 12.6	0.854
ASA score			0.140
I	4 (8.5%)	5 (6.4%)	
II	21 (44.7%)	47 (60.3%)	
III	20 (42.6%)	26 (33.3%)	
IV	2 (4.3%)	0	
HRD positive *	4 (8.5%)	13 (16.7%)	0.198
BRCA1/2 mutation ^†^	3 (6.4%)	7 (9.0%)	0.742
PARPi maintenance	3 (6.4%)	15 (19.2%)	0.048
Bevacizumab treatment	2 (4.3%)	4 (5.1%)	1.000
IDS	29 (61.7%)	66 (84.6%)	0.004
Histology			0.270
HGS	36 (76.6%)	69 (88.5%)	
Endometrioid	3 (6.4%)	1 (1.3%)	
Clear cell	4 (8.5%)	4 (5.1%)	
Other	4 (8.5%)	4 (5.1%)	
Stage			0.312
III	35 (74.5%)	64 (82.1%)	
IV	12 (25.5%)	14 (17.9%)	
CA-125 (IQR), U/mL	643 (243–1950)	670 (333–2128)	0.532
Additional surgical procedures ^‡^			
Diaphragm stripping	3 (6.4%)	3 (3.8%)	0.671
Splenectomy	2 (4.3%)	0	0.139
Rectosigmoid resection	2 (4.3%)	2 (2.6%)	0.631
Partial cystectomy	1 (2.1%)	2 (2.6%)	1.000

Data are presented as average ± SD, median (IQR) or number (percentage). * HRD status determined using the myChoice^®^ CDx assay (GIS ≥ 42); ^†^ includes pathogenic BRCA1/2 alteration detected (germline and/or somatic); ^‡^ extended surgical procedures performed in addition to standard cytoreduction. ASA—American Society of Anesthesiologists; BRCA—breast cancer gene; HGS—high-grade serous; HRD—homologous recombination deficiency; IDS—interval debulking surgery; PARPi—poly (ADP-ribose) polymerase inhibitors.

**Table 2:** Recurrence distribution and adverse events.

	Open (n = 47)	Robotic (n = 78)	*p*-Value
Intra-pelvic	21 (44.7%)	33 (42.3%)	0.795
Supra-pelvic	34 (72.3%)	45 (57.7%)	0.100
Retroperitoneal	16 (34.0%)	26 (33.3%)	0.935
Peritoneal and regional recurrence			
Intra-pelvic spleen	21 (44.7%)	33 (42.3%)	0.795
Supra-pelvic	34 (72.3%)	45 (57.7%)	0.100
Retroperitoneal	16 (34.0%)	26 (33.3%)	0.935
Parenchymal			
Liver	8 (17.0%)	10 (12.8%)	0.517
Spleen	5 (10.6%)	4 (5.1%)	0.295
Kidney	1 (2.1%)	0	0.376
Pancreas	0	1 (1.3%)	1.000
Extra abdominal			
Brain	1 (2.1%)	4 (5.1%)	0.649
Lungs	2 (4.3%)	5 (6.4%)	0.710
Psoas muscle	0	1 (1.3%)	1.000
Vaginal vault	0	1 (1.3%)	1.000
Abdominal wall *	3 (6.4%)	7 (9.0%)	0.742
Operative events			
Bleeding ^†^	1 (2.1%)	1 (1.3%)	1.000
Bladder injury	2 (4.3%)	2 (2.6%)	0.631
Bowel injury	0	1 (1.3%)	1.000
IVC injury	0	1 (1.3%)	1.000
Postop events ^‡^	4 (8.5%)	1 (1.3%)	0.066

Data are expressed as n (%).; IVC = inferior vena cava; * Among abdominal wall recurrences in the robotic group, one case represented a port-site recurrence; ^†^ bleeding necessitating transfusion > 2 units RBC; ^‡^ postoperative event = any eventration, MI, DVT, or wound infection within 30 days.

**Table 3:** Univariable and multivariable logistic regression analyses of factors associated with recurrence pattern after complete cytoreduction.

	Univariable OR (95% CI)	*p*-Value	Multivariable OR (95% CI)	*p*-Value
**Intra-Pelvic Recurrence**				
Robotic (vs. Open)	0.91 (0.44–1.88)	0.795	0.73 (0.33–1.61)	0.436
Age (per year)	1.02 (0.99–1.05)	0.199	1.02 (0.99–1.05)	0.195
ASA ≥ 3 (vs. <3)	0.90 (0.44–1.87)	0.785	0.70 (0.32–1.55)	0.379
IDS (yes vs. no)	1.83 (0.75–4.44)	0.183	1.90 (0.76–4.80)	0.172
PARPi (yes vs. no)	1.38 (0.51–3.75)	0.530	1.56 (0.54–4.51)	0.416
**Supra-Pelvic Recurrence**				
Robotic (vs. Open)	0.52 (0.24–1.14)	0.102	0.45 (0.20–1.05)	0.065
Age (per year)	1.00 (0.97–1.03)	0.976	1.00 (0.97–1.03)	0.823
ASA ≥ 3 (vs. <3)	1.28 (0.60–2.72)	0.526	1.10 (0.49–2.47)	0.826
IDS (yes vs. no)	1.39 (0.59–3.27)	0.452	1.70 (0.68–4.22)	0.256
PARPi (yes vs. no)	1.19 (0.42–3.43)	0.742	1.36 (0.45–4.13)	0.583
**Retro-Peritoneal Recurrence**				
Robotic (vs. Open)	0.97 (0.45–2.08)	0.935	1.00 (0.44–2.27)	0.997
Age (per year)	0.98 (0.95–1.02)	0.328	0.98 (0.95–1.01)	0.254
ASA ≥ 3 (vs. <3)	1.33 (0.62–2.83)	0.467	1.44 (0.64–3.25)	0.379
IDS (yes vs. no)	0.89 (0.37–2.14)	0.788	0.89 (0.36–2.23)	0.801
PARPi (yes vs. no)	1.31 (0.47–3.67)	0.608	1.22 (0.41–3.61)	0.715

Odds ratios (ORs) with 95% Wald confidence intervals (CIs). Univariable estimates derived from separate logistic regression models; multivariable estimates derived from a single model including all listed covariates. Reference categories were as follows: open surgery (vs. robotic), ASA < 3, primary debulking surgery (vs. IDS), and no PARP inhibitor maintenance. Age was modeled as a continuous variable (per year). ASA, American Society of Anesthesiologists; CI, confidence interval; IDS, interval debulking surgery; OR, odds ratio; PARP, poly(ADP-ribose) polymerase.

## Data Availability

The data presented in this study are available on request from the corresponding author due to institutional privacy regulations and patient confidentiality.

## Abbreviations

The following abbreviations are used in this manuscript:

ASA	American Society of Anesthesiologists
BRCA	Breast cancer gene
CA-125	Cancer antigen-125
DESKTOP	Descriptive Evaluation of preoperative Selection Criteria for Operability
DVT	Deep vein thrombosis
HGS	High-grade serous
HRD	Homologous recombination deficiency
IDS	Interval debulking surgery
IVC	Inferior vena cava
LANCE	Laparoscopic Cytoreduction after Neoadjuvant Chemotherapy in Ovarian Cancer
MIRRORS	Minimally Invasive Robotic Interval Debulking for Ovarian neoplasm
MIS	Minimally invasive surgery
NACT	Neoadjuvant chemotherapy
OC	Open cytoreduction
OS	Overall survival
PARP	Poly(ADP-ribose) polymerase
PARPi	Poly(ADP-ribose) polymerase inhibitor
PDS	Primary debulking surgery
DFS	Disease-free survival
RA-C	Robotic-assisted cytoreduction
R0	Complete macroscopic cytoreduction (no residual disease)
